# Magnetohydrodynamic Waves in an Asymmetric Magnetic Slab

**DOI:** 10.1007/s11207-017-1054-y

**Published:** 2017-02-01

**Authors:** Matthew Allcock, Robert Erdélyi

**Affiliations:** Solar Physics and Space Plasma Research Centre, School of Mathematics and Statistics, University of Sheffield, Hicks Building, Hounsfield Road, Sheffield, S3 7RH UK

**Keywords:** Coronal seismology, Magnetic fields, photosphere, Waves, magnetohydrodynamic, Waves, modes

## Abstract

Analytical models of solar atmospheric magnetic structures have been crucial for our understanding of magnetohydrodynamic (MHD) wave behaviour and in the development of the field of solar magneto-seismology. Here, an analytical approach is used to derive the dispersion relation for MHD waves in a magnetic slab of homogeneous plasma enclosed on its two sides by non-magnetic, semi-infinite plasma with different densities and temperatures. This generalises the classic magnetic slab model, which is symmetric about the slab. The dispersion relation, unlike that governing a symmetric slab, cannot be decoupled into the well-known sausage and kink modes, *i.e.* the modes have mixed properties. The eigenmodes of an asymmetric magnetic slab are better labelled as quasi-sausage and quasi-kink modes. Given that the solar atmosphere is highly inhomogeneous, this has implications for MHD mode identification in a range of solar structures. A parametric analysis of how the mode properties (in particular the phase speed, eigenfrequencies, and amplitudes) vary in terms of the introduced asymmetry is conducted. In particular, avoided crossings occur between quasi-sausage and quasi-kink surface modes, allowing modes to adopt different properties for different parameters in the external region.

## Introduction

Dynamic solar events have been widely observed to induce perturbations in the magnetically dominated coronal plasma (Banerjee *et al.*, 2007; McLaughlin, Hood, and de Moortel, 2011; Arregui, Oliver, and Ballester, 2012; Mathioudakis, Jess, and Erdélyi, 2013; Komm *et al.*, 2015). The inhomogeneous structuring of the plasma parameters determines the characteristics and speed of the waves propagating in these structures. The detection and analysis of waves provides an indirect way to gain information about the structure of the magnetic waveguide environment through the techniques of solar magneto-seismology, see *e.g.* reviews by Nakariakov and Verwichte (2005), Andries *et al.* (2009), Ruderman and Erdélyi (2009), De Moortel and Nakariakov (2012). Analytical models of waves in solar magnetic environments are crucial in the accurate employment of this technique.

Linear MHD waves that propagate along magnetic field and density stratifications have been widely researched in Cartesian geometry to gain first insight into the often complex magnetic structures, including *e.g.* wave propagation in sunspots, coronal loops, prominences, or coronal hole boundaries, to name a few applications. One of the simplest approaches is when a single planar magnetic and/or density interface is considered and the properties of the available surface waves that propagate along this interface are deduced (Roberts, 1981a). Adding a second planar interface to this model, in order to form a simple way of mimicking structuring, gives a magnetic slab where the external environment is not magnetised and has a uniform but different density from the internal slab density (Roberts, 1981b). Next, a homogeneous magnetic field may then be added to the external plasma (Edwin and Roberts, 1982), and the eigenvalues, *i.e.* allowed wave modes of a magnetised slab system, can be determined. After this initial surge in analytical linear models of solar atmospheric magnetic structures, Cartesian geometry was replaced in favour of cylindrical geometry, for which significant research has led to the development and applications of solar magneto-seismology (Rosenberg, 1970; Uchida, 1970; Zajtsev and Stepanov, 1975; Roberts, Edwin, and Benz, 1984; Goossens, Andries, and Aschwanden, 2002; see a review by Ruderman and Erdélyi, 2009) and hypothesised mechanisms for solar plasma heating including mechanisms such as phase mixing (Heyvaerts and Priest, 1983) and resonant absorption (see a review by Goossens, Erdélyi, and Ruderman, 2011, with plenty of references), to name a few. This trend is largely a result of ubiquitous observations of solar atmospheric magnetic flux tube oscillations, which lend themselves more naturally to cylindrical geometry.

The purpose of the present work is to generalise the isolated magnetic slab model by investigating the linear wave physics that arises when the density and temperature of one external plasma is different from that of the other, and external densities and temperatures are both different from those inside the slab. The interest in this generalisation comes from the asymmetry of the system, which gives rise to asymmetric quasi-sausage and quasi-kink eigenmodes that demonstrate mixed properties.

Magnetic structures in the corona where solar magneto-seismology has previously been employed (Nakariakov and Ofman, 2001; Nakariakov and Verwichte, 2005) are often better modelled by cylindrical than Cartesian geometry. Instead, structures closer to the photosphere, such as the magnetic canopy, provide an asymmetric slab structure for application of this work. The magnetic canopy is a region of dominant magnetic field parallel to the surface of the Sun between the much less magnetised photosphere and the chromosphere that connects the magnetic field lines between active regions. Another application may be to oscillations in magnetic bright points (MBPs). MBPs are often elongated vertical magnetic structures between granular cells of different densities and temperatures. Further information on lower atmospheric solar magneto-seismology can be found in *e.g.* de Pontieu and Erdélyi (2006).

First, a derivation of the dispersion relation is presented in Section 2. The eigenmode solutions of the dispersion relation are compared to those of a symmetric slab in Section 2.1. The properties of the waves in an incompressible and low-beta plasma are discussed in Sections 3.2 and 3.3. Wide and thin slab approximations are made in Sections 3.4 and 3.5. A numerical procedure is employed in Section 4 to investigate the effect that varying the external densities has on wave dispersion.

## Derivation of the Dispersion Relation

Consider an unbounded inviscid static plasma under an equilibrium magnetic field, $B(x)\mathbf{\widehat{z}}$, where $\mathbf{\widehat {z}}$ is the unit vector in the vertical direction, and where

1 $$ B(x)= \textstyle\begin{cases} B_{0} & \text{if }|x|\leq{x_{0}}, \\ 0 & \text{if }|x|>x_{0}, \end{cases}\displaystyle \quad\text{with $B_{0}$ constant.} $$

**Figure 1:**
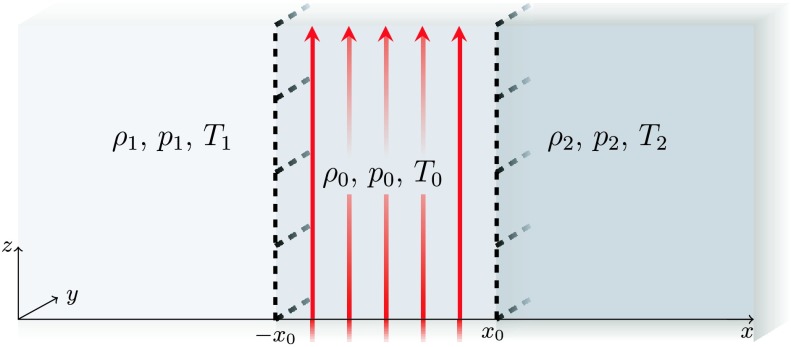
Equilibrium state inside the slab ($|x|\leq{}x_{0}$) and outside the slab ($x<-x_{0}$ and $x>x_{0}$). The red arrows illustrate the vertical magnetic field, $B(x)\mathbf{\widehat{z}}$, and the dashed black lines indicate the boundaries of the slab.

Within each region, the plasma is uniform and the equilibrium plasma pressure, density, and temperature are denoted by $p_{i}$, $\rho_{i}$, and $T_{i}$, respectively, for $i=0,1,2$ (see Figure 1). The effects of gravity are ignored throughout; it is important to note, however, that equilibrium density stratification itself in the solar atmosphere can be a consequence of gravity, but gravity can be ignored if the gravity scale height is large compared to the wavelength and the thickness of the magnetic slab, which is safe to assume for many small-scale solar atmospheric structures.

For the equilibrium conditions to be stable, there must be equilibrium pressure balance across each interface:

2 $$ p_{1}=p_{0}+\frac{B_{0}^{2}}{2\mu_{0}}=p_{2}. $$

Here, $\mu_{0}$ is the permeability of free space. The sound speed in each region is denoted by $c_{i}=\sqrt{\gamma{}p_{i}/\rho_{i}}$ for $i=0,1,2$, where $\gamma$ is the adiabatic index, which can be taken as constant across the whole domain if we take a single-fluid approximation in each region. Equilibrium pressure balance, Equation (2), gives the relationship between the external sound speeds and densities:

3 $$ \frac{c_{1}^{2}}{c_{2}^{2}}=\frac{\gamma{}p_{1}/\rho_{1}}{\gamma {}p_{2}/\rho_{2}}=\frac{\gamma{}p_{1}/\rho_{1}}{\gamma{}p_{1}/\rho _{2}}=\frac{\rho_{2}}{\rho_{1}}. $$

That is, to satisfy the equilibrium structure, the external densities and sound speeds must obey this relation.

The governing equations for the disturbance within the magnetic slab are the ideal MHD equations:

4 $$\begin{aligned} \rho\frac{\mathrm{d}\boldsymbol{v}}{\mathrm{d}t}&=-\nabla {p}-\frac {1}{\mu_{0}}\boldsymbol{B}\times( \nabla\times\boldsymbol{B}), \end{aligned}$$

5 $$\begin{aligned} \frac{\partial\rho}{\partial{t}}+\nabla\cdot(\rho\boldsymbol {v})&=0, \end{aligned}$$

6 $$\begin{aligned} \frac{\mathrm{d}}{\mathrm{d}t} \biggl( \frac{p}{\rho^{\gamma }} \biggr) &=0, \end{aligned}$$

7 $$\begin{aligned} \frac{\partial{\boldsymbol{B}}}{\partial{t}}&=\nabla\times (\boldsymbol{v}\times\boldsymbol{B}), \end{aligned}$$

where the variables $\boldsymbol{v}=(v_{x},v_{y},v_{z})$, $\boldsymbol {B}$, $p$, and $\rho$ are the velocity, magnetic field, pressure, and density, at time $t$.

After linearising about a static basic state, we can combine the governing equations and seek solutions of the form

8 $$ v_{x}(\boldsymbol{x},t)=\widehat{v}_{x}(x) \mathrm{e}^{i(kz-\omega {t})}, \qquad v_{y}(\boldsymbol{x},t)=0, \qquad v_{z}(\boldsymbol{x},t)=\widehat{v}_{z}(x) \mathrm{e}^{i(kz-\omega{t})}, $$

where $k$ is the component of the wavenumber vector in the $\mathbf {\widehat{z}}$-direction and $\omega$ is the angular frequency. This restricts the investigation to waves propagating parallel to the equilibrium magnetic field, with velocity perturbation amplitude $\widehat{v}_{x}(x)$ in the $\mathbf{\widehat{x}}$-direction, and $\widehat{v}_{z}(x)$ in the $\mathbf{\widehat{z}}$-direction. With this ansatz, the linearised equations reduce to

9 $$\begin{aligned} &-\omega^{2}\widehat{v}_{x}=\bigl(c_{0}^{2}+v_{\mathrm{A}}^{2} \bigr) \bigl(\widehat {v}_{x}^{\,\prime\prime}+ik \widehat{v}_{z}^{\,\prime}\bigr)-v_{\mathrm{A}}^{2}ik \widehat {v}_{z}^{\,\prime}-v_{\mathrm{A}}^{2}k^{2} \widehat{v}_{x}, \end{aligned}$$

10 $$\begin{aligned} &-\omega^{2}\widehat{v}_{z}=c_{0}^{2}ik \bigl(\widehat{v}_{x}^{\,\prime}+ik\widehat {v}_{z} \bigr), \end{aligned}$$

where $v_{\mathrm{A}}=B_{0}/\sqrt{\rho_{0}\mu}$ is the Alfvén speed and $'=\mathrm{d}/\mathrm{d}x$. These equations can be combined to give an ordinary differential equation for $\widehat{v}_{x}$, namely

11 $$ \widehat{v}_{x}^{\,\prime\prime}-m_{0}^{2} \widehat{v}_{x}=0, $$

where

12 $$ m_{0}^{2}=\frac{(k^{2}v_{\mathrm{A}}^{2}-\omega ^{2})(k^{2}c_{0}^{2}-\omega^{2})}{(c_{0}^{2}+v_{\mathrm {A}}^{2})(k^{2}c_{\mathrm{T}}^{2}-\omega^{2})}, \qquad c_{\mathrm {T}}^{2}= \frac{c_{0}^{2}v_{\mathrm{A}}^{2}}{c_{0}^{2}+v_{\mathrm{A}}^{2}}, $$

which is valid for perturbations inside the slab. This is identical to the corresponding equation for a symmetric slab derived by Roberts (1981b). By setting $v_{\mathrm{A}}=0$, a change of subscripts in Equation (11) is all that is required to derive the ordinary differential equation governing linear perturbations outside the slab, namely

13 $$ \widehat{v}_{x}^{\,\prime\prime}-m_{j}^{2} \widehat{v}_{x}=0, $$

where

14 $$ m_{j}^{2}=k^{2}-\frac{\omega^{2}}{c_{j}^{2}}, \quad \text{for $j=1,2$.} $$

The solutions of Equations (11) and (13) are a linear combination of the hyperbolic functions. We restrict our model to waves trapped by the slab by ensuring $\widehat{v}_{x}\to{0}$ as $|x|\to {\infty}$. The general solution for the velocity perturbation in the $\mathbf{\widehat{x}}$-direction is

15 $$ \widehat{v}_{x}(x)= \textstyle\begin{cases} A(\cosh{m_{1}x}+\sinh{m_{1}x}), & \text{if }x< -x_{0}, \\ B\cosh{m_{0}x}+C\sinh{m_{0}x}, & \text{if }|x|\leq{x_{0}}, \\ D(\cosh{m_{2}x}-\sinh{m_{2}x}), & \text{if }x>x_{0}, \end{cases} $$

where $A$, $B$, $C$, and $D$ are arbitrary constants (with respect to $x$). The plasma pressure perturbation can be assumed to be of the form $p(\boldsymbol{x},t)=\widehat{p}(x)\mathrm{e}^{i(kz-\omega{t})}$, then the total pressure perturbation amplitude across the whole domain is

16 $$ \widehat{P}(x)=\widehat{v}_{x}^{\,\prime}(x) \textstyle\begin{cases} \varLambda_{1}/m_{1}, & \text{if } x< -x_{0}, \\ \varLambda_{0}/m_{0}, & \text{if }|x|\leq{}x_{0}, \\ \varLambda_{2}/m_{2}, & \text{if }x>x_{0}, \end{cases} $$

where

17 $$ \varLambda_{0}=-\frac{i\rho_{0}(k^{2}v_{\mathrm{A}}^{2}-\omega ^{2})}{m_{0}\omega}, \qquad \varLambda_{1}= \frac{i\rho_{1}\omega }{m_{1}}, \quad\text{and} \quad\varLambda_{2}=\frac{i\rho_{2}\omega }{m_{2}}. $$

The boundary conditions are continuity of velocity and total pressure across the slab boundaries at $x=\pm{x_{0}}$, which gives the four coupled homogeneous algebraic equations

18 $$ \left ( \begin{matrix} c_{1}-s_{1} &-c_{0} &s_{0} &0 \\ 0 &c_{0} &s_{0} &s_{2}-c_{2} \\ \varLambda_{1}(c_{1}-s_{1}) &\varLambda_{0}s_{0} &-\varLambda_{0}c_{0} &0 \\ 0 &\varLambda_{0}s_{0} &\varLambda_{0}c_{0} &-\varLambda_{2}(s_{2}-c_{2}) \end{matrix} \right ) \left ( \begin{matrix} A \\ B \\ C \\ D \end{matrix} \right ) = \left ( \begin{matrix} 0 \\ 0 \\ 0 \\ 0 \end{matrix} \right ) , $$

where $c_{i}=\cosh{m_{i}x_{0}}$ and $s_{i}=\sinh{m_{i}x_{0}}$ for $i=0,1,2$, for brevity. The condition for the existence of non-trivial solutions to this system of equations is that the determinant of the above matrix of coefficients is zero. Applying this condition gives us the dispersion relation for an asymmetric slab, namely

19 $$ 2\bigl(\varLambda_{0}^{2}+\varLambda_{1} \varLambda_{2}\bigr)+\varLambda_{0}(\varLambda _{1}+ \varLambda_{2}) ( \tanh{m_{0}x_{0}}+ \coth{m_{0}x_{0}} ) =0. $$

Using the notation introduced by Equation (14), we arrive at

20 $$\begin{aligned} &\omega^{4}m_{0}^{2}+\frac{\rho_{0}}{\rho_{1}}m_{1} \frac{\rho _{0}}{\rho_{2}}m_{2}\bigl(k^{2}v_{\mathrm{A}}^{2}- \omega^{2}\bigr)^{2} \\ &\quad {}-\frac{1}{2}m_{0}\omega^{2}\bigl(k^{2}v_{\mathrm{A}}^{2}- \omega ^{2}\bigr) \biggl( \frac{\rho_{0}}{\rho_{1}}m_{1}+ \frac{\rho_{0}}{\rho _{2}}m_{2} \biggr) ( \tanh{m_{0}x_{0}}+ \coth{m_{0}x_{0}} ) =0. \end{aligned}$$

### Comparison with a Symmetric Slab

There is an intrinsic difference between perturbations along symmetric and asymmetric magnetic slabs. The dispersion relation governing an asymmetric slab is a single equation, whereas the dispersion relation governing a symmetric slab (Roberts, 1981a) consists of two independent equations, corresponding to the sausage and kink eigenmodes.

Under the approximation that the densities and temperatures of the external plasma are of the same order with respect to each other, the dispersion relation, Equation (20), can be factorised to give the approximate dispersion relation

21 $$ \bigl[ \varLambda_{0}(\varLambda_{1}+\varLambda_{2})+2 \varLambda_{1}\varLambda _{2}\tanh{m_{0}x_{0}} \bigr] \bigl[ \varLambda_{0}(\varLambda _{1}+\varLambda _{2})+2\varLambda_{1}\varLambda_{2}\coth{m_{0}x_{0}} \bigr] =0. $$

The expressions for the variables $\varLambda_{i}$ for $i=0,1,2$ in Equations (17) can be employed to yield the approximately symmetric dispersion relation

22 $$ \bigl(k^{2}v_{\mathrm{A}}^{2}-\omega^{2}\bigr) \biggl( \frac{\rho_{0}}{\rho _{1}}m_{1}+\frac{\rho_{0}}{\rho_{2}}m_{2} \biggr) =2\omega ^{2}m_{0}\left ( \hspace{-0.06in} \begin{matrix}&\tanh\\ &\coth \end{matrix} \right ) (m_{0}x_{0}). $$

This equation is now in an analogous form to the dispersion relation corresponding to MHD waves along a symmetric magnetic slab (Roberts, 1981b), namely

23 $$ \bigl(k^{2}v_{\mathrm{A}}^{2}-\omega^{2}\bigr) \frac{\rho_{0}}{\rho_{\textrm {e}}}m_{\mathrm{e}}=\omega^{2}m_{0}\left ( \hspace{-0.06in} \begin{matrix}&\tanh\\ &\coth \end{matrix} \right ) (m_{0}x_{0}), $$

where external parameters are denoted by subscript $\mathrm{e}$.

### Asymmetric Eigenmodes

There is a rich spectrum of MHD waves supported by a magnetic slab. In a symmetric slab, the dispersion relation, Equation (23), consists of two decoupled equations that correspond to the two types of fundamental wave supported by the slab: the “sausage” and “kink” MHD waves. Sausage and kink modes have been observed, for example, in chromospheric fibrils, where the ubiquity of these waves has been linked to coronal heating (Morton *et al.*, 2012).

Sausage and kink modes can be further categorised into “surface” and “body” modes. Surface modes are waves more enhanced at the slab boundaries, whereas body waves are characterised by oscillations permeating spatially throughout the slab, having their maximum amplitude within the slab. Mathematically, surface waves correspond to exponential solutions of Equation (11), that is, they exist when $m_{0}^{2}>0$, which occurs when the phase speed $\omega/k$ satisfies

24 $$ \frac{\omega}{k}< c_{\mathrm{T}} \quad\text{or} \quad\text{min}\{ c_{0}, v_{\mathrm{A}}\}< \frac{\omega}{k}< \text{max} \{c_{0}, v_{\textrm {A}}\}. $$

Body waves correspond to spatially oscillatory solutions (*i.e.* they can have nodes inside the slab), that is, they exist when $m_{0}^{2}<0$, which occurs when

25 $$ c_{\mathrm{T}}< \frac{\omega}{k}< \text{min}\{c_{0}, v_{\mathrm{A}}\} \quad\text{or} \quad\text{max}\{c_{0}, v_{\mathrm{A}}\}< \frac {\omega }{k}. $$

**Figure 2:**
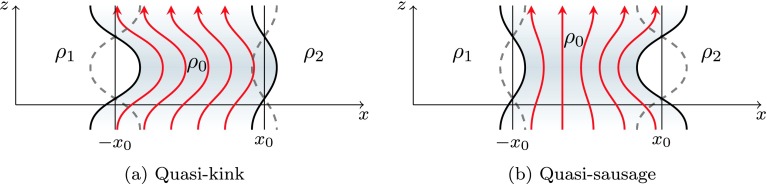
Quasi-kink and quasi-sausage modes with external density ordering $\rho_{1}>\rho_{2}$. The red lines illustrate the perturbed magnetic field, the thick solid black lines illustrate the perturbed slab boundaries, and the dashed lines illustrate the future position of the slab boundaries after half a period.

For an asymmetric slab, the sausage and kink modes are modified by the external density difference, causing an asymmetry of the oscillation amplitude on each side of the slab (for visualisation see Figures 2a and 2b). We call these asymmetric eigenmodes quasi-sausage and quasi-kink modes. In a symmetric slab, sausage modes are characterised by a line of zero perturbation at the centre of the slab. In an asymmetric slab, this line is shifted towards the side of greatest external density. For symmetric kink modes, the width of the perturbed slab remains constant along the slab, but this characteristic is dropped in an asymmetric slab. This highlights the mixed nature of these modes.

**Figure 3:**
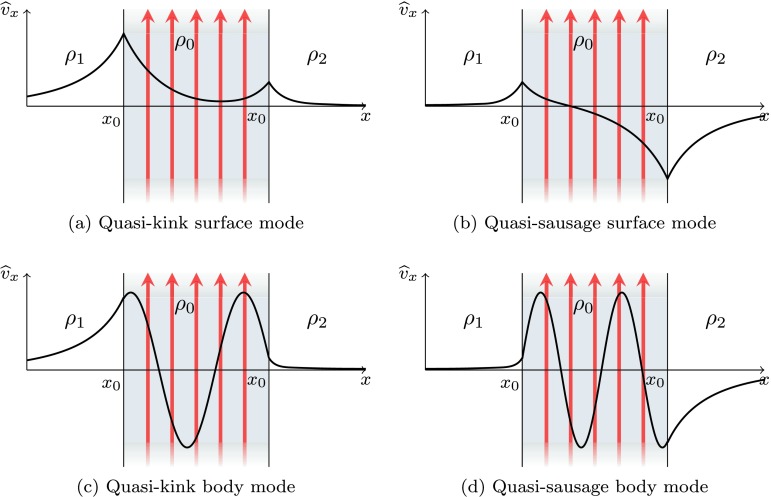
The transverse velocity perturbation amplitude, $\widehat {v}_{x}$ as a function of the transverse spatial coordinate, $x$, for quasi-sausage and quasi-kink modes in an isolated magnetic slab with external density ordering $\rho_{1}>\rho_{2}$.

The surface and body properties also take a modified form in an asymmetric slab. For a surface mode (for visualisation see Figures 3a and 3b), the wave power distribution across the slab has a single minimum. The displacement of this minimum from the centre of the slab is a consequence of the asymmetry in the external plasma. The intensity of the maximum amplitudes on the left and right boundaries of the slab ($x=\pm {}x_{0}$) is different, reflecting the asymmetry in the external plasma.

Body modes are also affected by the asymmetric external environment (for visualisation see Figures 3c and 3d). Local maxima and minima in wave power are shifted towards the external plasma of higher density for a quasi-kink body mode and towards the external plasma of lower density for a quasi-sausage mode. However, body modes depend only weakly on the external plasma parameters and are therefore less affected than surface modes. This is shown analytically in Sections 3.4 and 3.5.

## Analytical Solutions

In this section, simplifications are made to the dispersion relation, Equation (20), and approximate dispersion relation, Equation (22). Thin slab, wide slab, low-beta, and incompressible approximations are made.

### Spurious Solutions

The solution to the exact and approximate dispersion relations, Equations (20) and (22), given by $\omega =kv_{\mathrm{A}}$, is spurious and does not correspond to an eigenmode. To see this, observe that $m_{0}=0$ for this solution, which leads to a linear (rather than oscillatory or exponential) solution to the governing differential equation, Equation (2.20). The same can be said for the solutions $\omega=kc_{0}$ and $\omega=kc_{\mathrm{T}}$. This rules out the possibility of the existence of pure sound waves and pure Alfvén waves.

### Incompressible Approximation

Compressibility is essential for the propagation of sound waves. Consider the dispersion relation, Equation (20), in the limit of incompressibility, that is, when $\gamma\to\infty$, where $\gamma$ is the adiabatic index. In this limit, the sound speeds become unbounded, therefore the tube speed behaves like $c_{\mathrm{T}}\to {v_{\mathrm{A}}}$. This means that $m_{j}\to{k}$ for $j=0,1,2$, so that the dispersion relation reduces to

26 $$\begin{aligned} &\omega^{4} +\frac{\rho_{0}^{2}}{\rho_{1}\rho _{2}}\bigl(k^{2}v_{\mathrm {A}}^{2}- \omega^{2}\bigr)^{2} \\ &\quad {}-\frac{1}{2}\omega^{2}\bigl(k^{2}v_{\mathrm{A}}^{2}- \omega^{2}\bigr) \biggl( \frac{\rho_{0}}{\rho_{1}}+\frac{\rho_{0}}{\rho_{2}} \biggr) ( \tanh{kx_{0}}+\coth{kx_{0}} ) =0. \end{aligned}$$

This is a special case of the dispersion relation previously derived by Ruderman (1992), who found solitons propagating on a system of $N$ tangential discontinuities. This is a quadratic equation in $\omega^{2}$, with solutions given by

27 $$ \omega^{2}=\frac{1}{2}k^{2}v_{\mathrm{A}}^{2} \biggl[ \frac{2+\sigma \pm \sqrt{\sigma^{2}-4\frac{\rho_{1}\rho_{2}}{\rho _{0}^{2}}}}{1+\sigma +\frac{\rho_{1}\rho_{2}}{\rho_{0}^{2}}} \biggr] , $$

where

28 $$ \sigma=\frac{1}{2} \biggl( \frac{\rho_{1}}{\rho_{0}}+\frac{\rho _{2}}{\rho_{0}} \biggr) ( \tanh{kx_{0}}+\coth{kx_{0}} ) . $$

These solutions hold for all $kx_{0}$ and describe surface modes with sub-Alfvénic phase speed.

**Figure 4:**
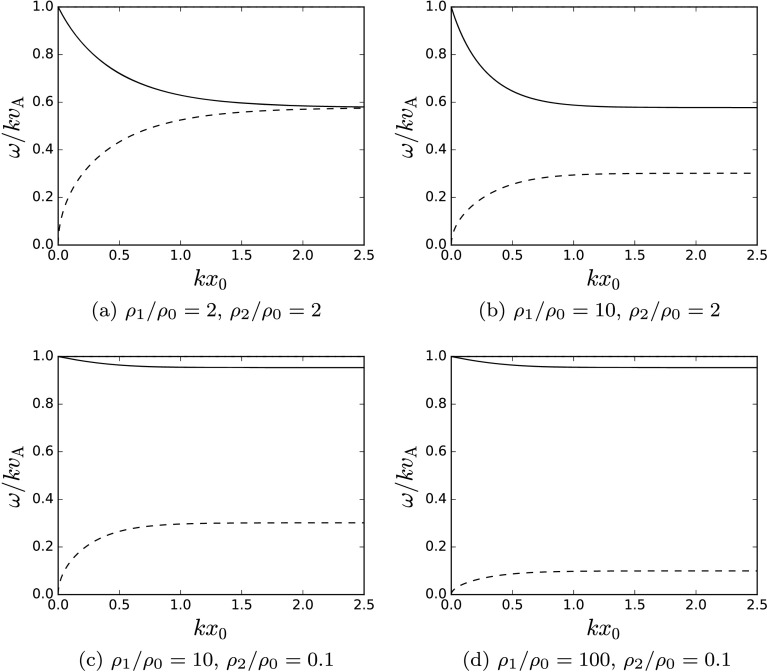
The behaviour of the modes in an incompressible slab. The fast surface modes and all the body modes degenerate, leaving two sub-Alfvénic surface modes. (a) A symmetric slab, (b) – (d) asymmetric slabs.

Figures 4a – 4d demonstrate that in a thin incompressible slab, the phase speeds of these modes approaches zero or the Alfvén speed. In a symmetric wide incompressible slab, the phase speeds converge to the same speed (Figure 4a), whereas in an asymmetric slab, the phase speeds converge to different speeds (Figures 4b – 4d) that depend upon the values of the external densities. This observation is mirrored by both fast and slow surface modes in the more general solutions of a compressible slab solved numerically to give Figure 7a.

### Low-Beta Approximation

The following section concerns the case when the magnetic pressure strongly dominates the gas pressure within the slab, *i.e.* $\beta:=2\mu_{0}{p_{0}}/B_{0}^{2}\ll1$. This is known as the low-beta approximation and corresponds to the Alfvén speed dominating the sound speed in the slab; this provides a good approximation of the solar coronal environment. However, for application here an external magnetic field needs to be considered; this is the subject of a follow-up article.

Under this speed ordering, $m_{0}^{2}\approx{}k^{2}-\omega ^{2}/v_{\mathrm{A}}^{2}$. After a numerical investigation, it is clear that the frequency of waves in this approximation satisfies $\omega ^{2}\ll{}k^{2}v_{\mathrm{A}}^{2}$, in which case $m_{0}^{2}\approx k^{2}$ provides a valid approximation. This means that $m_{0}^{2}>0$ and the solutions are surface modes. For a symmetric slab of low-beta plasma (*e.g.* Roberts 1981b), the dispersion relation reduces to a quadratic expression in $\omega^{2}$ whose solutions are the fast sausage and kink surface modes given by

29 $$ \omega^{2}=k^{2}c_{\mathrm{e}}^{2}\left ( \frac{\sqrt{1+\gamma ^{2}\left ( \begin{matrix}&\tanh^{2} \\&\coth^{2} \end{matrix} \right ) (kx_{0})}-1} {\frac{1}{2}\gamma^{2}\left ( \begin{matrix}&\tanh^{2} \\&\coth^{2} \end{matrix} \right ) (kx_{0})} \right ) , $$

where $c_{\mathrm{e}}$ is the external sound speed, along with a spurious solution.

Unfortunately, for the more general case of an asymmetric slab of low-beta plasma, the dispersion relation does not reduce to an analytically solvable equation. However, we find numerically that there are two fast surface modes. The quasi-sausage surface mode is not present for small $kx_{0}$, but becomes a solution at an intermediate value of $kx_{0}$ with phase speed $\omega^{2}/k^{2}=\min {(c_{1}^{2},c_{2}^{2})}$. The quasi-kink surface mode is present for all values of $kx_{0}$. Qualitatively, the solutions for a low-beta plasma are analogous to the fast quasi-sausage and quasi-kink mode solutions, discussed later in Sections 3.4 and 3.5.

In the following section, thin and wide slab approximations are made to the approximate dispersion relation, Equation (22), rather than the exact dispersion relation, Equation (20). Here, the aim is to retrieve the variety of wave modes with future applications in mind.

### Thin Slab Approximation

Consider the case where the wavelength, $\lambda$, of the waves propagating in the system is much greater than the width of the slab, $2x_{0}$, *i.e* $x_{0}/\lambda=kx_{0}/2\pi\ll1$.

First, consider the quasi-sausage surface modes, which are governed by the tanh version of Equation (22), for $m_{0}^{2}>0$. In the thin slab limit, this equation reduces to

30 $$ \bigl(k^{2}v_{\mathrm{A}}^{2}-\omega^{2}\bigr) \biggl( \frac{\rho_{0}}{\rho _{1}}m_{1}+\frac{\rho_{0}}{\rho_{2}}m_{2} \biggr) =2\omega ^{2}m_{0}^{2}x_{0}. $$

Clearly, $\omega^{2}={k^{2}v_{\mathrm{A}}^{2}}$ is a solution, but as previously noted, this is spurious. The other solution for $\omega ^{2}$ behaves like $\omega^{2}\to{}k^{2}c_{\mathrm{T}}^{2}$ as $kx_{0}\to{0}$. To first order in $kx_{0}$, this solution is a slow quasi-sausage surface mode given by

31 $$ \omega^{2}=k^{2}c_{\mathrm{T}}^{2} \biggl[ 1- \frac {2(kx_{0})(c_{0}^{2}-c_{\mathrm{T}}^{2})}{(c_{0}^{2}+v_{\textrm {A}}^{2}) ( \frac{\rho_{0}}{\rho_{1}}\frac {(c_{1}^{2}-c_{\textrm {T}}^{2})^{1/2}}{c_{1}}+\frac{\rho_{0}}{\rho_{2}}\frac {(c_{2}^{2}-c_{\mathrm{T}}^{2})^{1/2}}{c_{2}} ) } \biggr] , $$

which is less than $k^{2}c_{\mathrm{T}}^{2}$ and exists only when $c_{1}>c_{\mathrm{T}}$ and $c_{2}>c_{\mathrm{T}}$.

**Figure 5:**
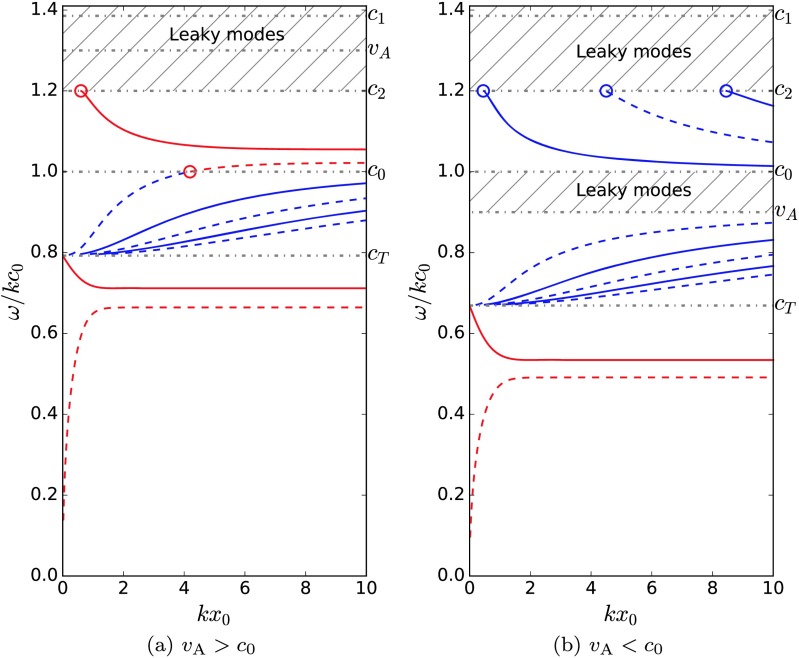
Dispersion diagram for the dispersion relation, Equation (20). The surface (body) modes are in plotted red (blue), and the sausage (kink) modes are represented by solid (dashed) lines. The density ratios are $\rho_{1}/\rho_{0} = 1.5$ and $\rho_{2}/\rho_{0} = 2$, and the characteristic speed orderings are $c_{2}=1.2c_{0}$ and (a) $v_{\mathrm{A}}=1.3c_{0}$ and (b) $v_{\mathrm{A}}=0.9c_{0}$.

It is interesting to note that if $c_{1}=c_{2}=c_{\mathrm{e}}$ (and therefore $\rho_{1}=\rho_{2}=\rho_{\mathrm{e}}$ by Equation (3)), then there exists a second solution to Equation (30). By letting $\omega^{2}=k^{2}c_{\textrm {e}}^{2}(1+\nu)$ for some $\nu\ll1$ in Equation (30), we find the solution

32 $$ \omega^{2}=k^{2}c_{\mathrm{e}}^{2} \biggl( 1- \biggl[ \frac{\rho _{\textrm {e}}}{\rho_{0}}\frac{c_{\mathrm{e}}^{2}(c_{0}^{2}-c_{\textrm {e}}^{2})(kx_{0})}{(c_{0}^{2}+v_{\mathrm{A}}^{2})(c_{\textrm {T}}^{2}-c_{\mathrm{e}}^{2})} \biggr] ^{2} \biggr) $$

in the thin slab limit. This is a fast sausage surface mode, and it degenerates (as a solution in the thin slab limit) as $c_{1}$ and $c_{2}$ become distinct, but this mode can still exist with a phase speed below the cut-off at $\min(c_{1}, c_{2})$. By solving the dispersion relation numerically, it can be shown that although the fast quasi-sausage surface mode does not exist in the thin slab limit for an asymmetric slab, it can exist in the wide slab limit (*i.e.* as a solution to Equation (36)) when $c_{0}< v_{\mathrm{A}}$, $c_{1}< v_{\mathrm{A}}$, and $c_{2}< v_{\mathrm{A}}$. For example, in Figure 5a, the minimum of $c_{1}$ and $c_{2}$ becomes a new cut-off, causing the fast quasi-sausage surface mode to degenerate for small $kx_{0}$.

Next, consider quasi-kink surface mode solutions in the thin slab limit, which are governed by the coth version of Equation (22), for $m_{0}^{2}>0$. As $kx_{0}\to{}0$, we have $m_{0}x_{0}\to{}0$, so $\coth{m_{0}x_{0}}\to{}1/m_{0}x_{0}$. This simplifies the dispersion relation to

33 $$ \omega^{2}=\frac{k^{2}x_{0}v_{\mathrm{A}}^{2} ( \frac{\rho _{0}}{\rho_{1}}m_{1}+\frac{\rho_{0}}{\rho_{2}}m_{2} ) }{ ( \frac{\rho_{0}}{\rho_{1}}m_{1}x_{0}+\frac{\rho_{0}}{\rho _{2}}m_{2}x_{0} ) +2}\approx{}\frac{1}{2}k^{2}v_{\textrm {A}}^{2} \biggl( \frac{\rho_{0}}{\rho_{1}}+\frac{\rho_{0}}{\rho _{2}} \biggr) kx_{0}. $$

This is a slow quasi-kink surface mode that behaves like $\omega/k\to {0}$ in the thin slab limit.

For body waves in the thin slab approximation, following the same procedure as for surface waves turns out to be fruitless, so we must reconsider our assumptions. Unfortunately, letting $m_{0}x_{0}\to{}0$ as $kx_{0}\to{}0$, whilst valid for surface modes, does not exhaust all of the modes of the slab. Instead, we must consider the scenario where $m_{0}x_{0}$ remains finite as $kx_{0}\to0$. This can occur only if $|m_{0}^{2}|\to\infty$ as $kx_{0}\to0$. To ensure $|m_{0}^{2}|\to \infty$, we are restricted to solutions that behave like $\omega ^{2}\to{}k^{2}c_{\mathrm{T}}^{2}$ as $kx_{0}\to0$. Upon consideration of Equation (22), this can only be the case when $m_{0}^{2}<0$, *i.e.* only for body modes. To find these solutions, set $\omega^{2}=k^{2}c_{\mathrm{T}}^{2}(1+\nu (kx_{0})^{2})$ for some $\nu>0$ that is to be determined. To see why this form has been chosen, a substitution into the definition of $m_{0}^{2}$ demonstrates that $|m_{0}^{2}|\to\infty$ and $m_{0}x_{0}$ remains bounded as $kx_{0}\to0$, as required. Using this ansatz, Equation (22) has a countably infinite set of quasi-sausage body solutions, which in the thin slab limit, behave like

34 $$ \omega^{2}=k^{2}c_{\mathrm{T}}^{2} \biggl( 1+ \frac{c_{\textrm {T}}^{4}(kx_{0})^{2}}{c_{0}^{2}v_{\mathrm{A}}^{2}\pi^{2}j^{2}} \biggr) , \quad j=1,2,\ldots. $$

There are also quasi-kink body solutions that in the thin slab limit behave like

35 $$ \omega^{2}=k^{2}c_{\mathrm{T}}^{2} \biggl( 1+ \frac{c_{\textrm {T}}^{4}(kx_{0})^{2}}{c_{0}^{2}v_{\mathrm{A}}^{2}\pi^{2}(j-\frac {1}{2})^{2}} \biggr) , \quad j=1,2,\ldots. $$

Equations (34) and (35) show us that to quadratic order in $kx_{0}$, the quasi-sausage and quasi-kink body modes do not depend on the external environment parameters. The effects of external density and temperature are felt in the higher order terms, which explains why Equations (34) and (34) are identical to the corresponding solutions in a thin symmetric slab derived by Roberts (1981b).

### Wide Slab Approximation

We now turn our attention to the behaviour of solutions to the dispersion relation in the wide slab limit, $kx_{0}\gg{1}$. As the width of the slab increases with respect to the wavelength, the slab boundaries have diminishing effect on the modes and each other.

The supposition that $m_{0}x_{0}\gg{1}$ when $kx_{0}\gg{1}$ (which is verified by Roberts, 1981b) leads to the approximation $\tanh {m_{0}x_{0}}\approx1$, which reduces the dispersion relation, Equation (22), to

36 $$ \bigl(k^{2}v_{\mathrm{A}}^{2}-\omega^{2}\bigr) \biggl( \frac{\rho_{0}}{\rho _{1}}m_{1}+\frac{\rho_{0}}{\rho_{2}}m_{2} \biggr) =2\omega^{2}m_{0}. $$

A comparison with Roberts (1981a) demonstrates that in the wide slab limit, the dispersion relation reduces to the single interface problem, but with a modified density ratio and wavenumber. This does not qualitatively change the solutions.

Unfortunately, the body waves have no parallel in the single interface model because body waves in a slab that are externally evanescent owe their existence to the two interfaces. For body modes in the wide slab limit, there exists a solution that behaves like $\omega^{2}\to {}k^{2}c_{0}^{2}$ as $kx_{0}\to\infty$. To see this, substitute the ansatz $\omega^{2}=k^{2}c_{0}^{2} ( 1+\nu/(kx_{0})^{2} ) $ into the dispersion relation to retrieve the family of quasi-sausage body modes given by

37 $$ \omega^{2}=k^{2}c_{0}^{2} \biggl( 1- \frac{\pi^{2}(j-\frac {1}{2})^{2}c_{0}^{2}}{(v_{\textrm {A}}^{2}-c_{0}^{2})(kx_{0})^{2}} \biggr) , \quad j=1,2,\ldots $$

in the wide slab limit. Similarly, there exist quasi-kink body mode solutions given by

38 $$ \omega^{2}=k^{2}c_{0}^{2} \biggl( 1- \frac{\pi ^{2}j^{2}c_{0}^{2}}{(v_{\mathrm{A}}^{2}-c_{0}^{2})(kx_{0})^{2}} \biggr) , \quad j=1,2,\ldots $$

in the wide slab limit. These are valid solutions for the characteristic speed ordering $v_{\mathrm{A}}>c_{0}$.

This analysis may then be conducted for $v_{\mathrm{A}}< c_{0}$ to find that in the wide slab limit, there exist quasi-sausage body mode solutions given by

39 $$ \omega^{2}=k^{2}v_{\mathrm{A}}^{2} \biggl( 1- \frac{\pi^{2}(j-\frac {1}{2})^{2}v_{\mathrm{A}}^{2}}{(c_{0}^{2}-v_{\textrm {A}}^{2})(kx_{0})^{2}} \biggr) , \quad j=1,2,\ldots $$

and quasi-kink body mode solutions of the form

40 $$ \omega^{2}=k^{2}v_{\mathrm{A}}^{2} \biggl( 1- \frac{\pi ^{2}j^{2}v_{\mathrm{A}}^{2}}{(c_{0}^{2}-v_{\textrm {A}}^{2})(kx_{0})^{2}} \biggr) , \quad j=1,2,\ldots. $$

These solutions to the approximate asymmetric slab dispersion relation demonstrate that to inverse quadratic order of $kx_{0}$, the wide slab body modes are independent of the external plasma parameters. Therefore, Equations (37), (38), (39), and (40) are identical to the body mode solutions in a wide symmetric slab (Roberts, 1981b). Equations (31) – (35), (37) and (38) also appear in Li, Habbal, and Chen (2013) for a symmetric magnetic slab with shear flow when the shear flow speed is set to zero.

## Numerical Solutions

In this section, the asymmetric dispersion relation is solved numerically, with particular interest placed on the effect of changing the ratio of the external densities.

### Density Ratio Variation

**Figure 6:**
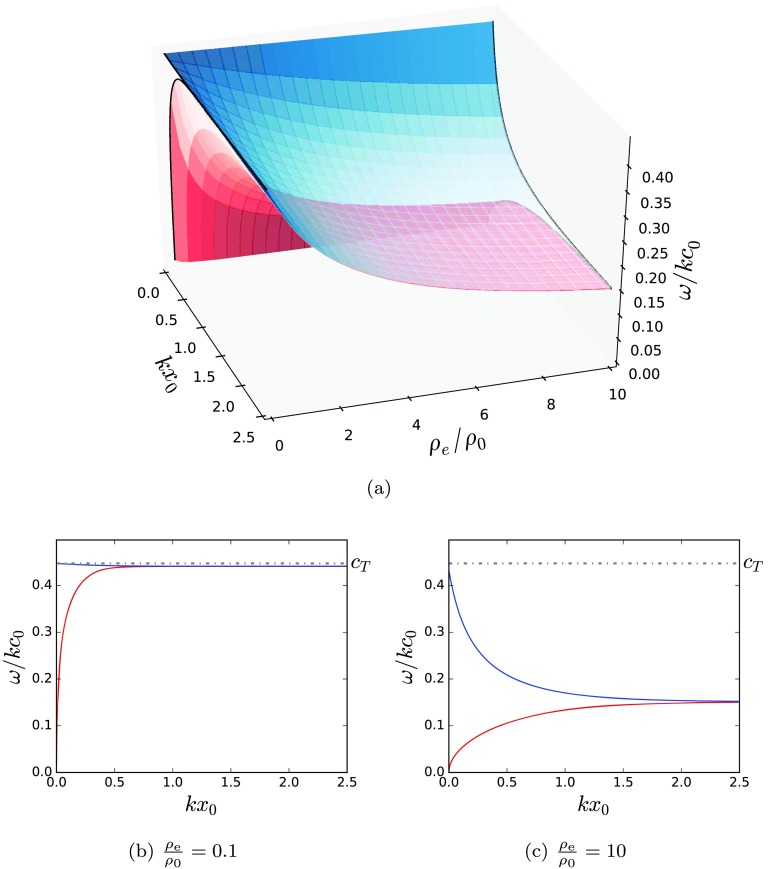
The effect of varying the ratio, $\rho_{\mathrm{e}}/\rho _{0}$, of the slab density to the symmetric external density, on the dispersion of the slow surface modes of a magnetic slab in a symmetric external plasma. The red and blue surfaces correspond to the kink and sausage modes, respectively. Panels (b) and (c) are slices of panel (a) at specific values of $\rho_{\mathrm{e}}/\rho_{0}$. These slices are superimposed onto panel (a) as black lines. The characteristic speed orderings are $v_{\mathrm{A}}=0.5c_{0}$, $c_{\mathrm{e}}=c_{0}$.

First, consider a magnetised slab with symmetric non-magnetic external plasma, as described by Roberts (1981b). Figures 6a – 6c illustrate how varying the ratio of external to internal density affects the propagation speeds of the slow kink and sausage surface modes. An increase in the density ratio, $\rho_{\mathrm{e}}/\rho_{0}$, causes a decrease in the propagation speed of the slow modes. The fast surface modes demonstrate an identical behaviour (not shown). The body modes are weakly dependent on the external density, so that the propagation speed decreases only negligibly as $\rho_{\mathrm{e}}/\rho_{0}$ increases.

More generally, consider an asymmetric slab whose equilibrium conditions are given by Figure 1. In Section 2.1 it was shown that the dispersion relation does not decouple into separate sausage and kink mode equations. However, their characteristics remain by and large, therefore the labels of “quasi-sausage” and “quasi-kink” may be used by referring to the anti-phase and in-phase oscillatory behaviour of the slab boundaries. However, we note that the quasi-kink mode now does not retain the width of the perturbed slab like the symmetric kink mode does.

**Figure 7:**
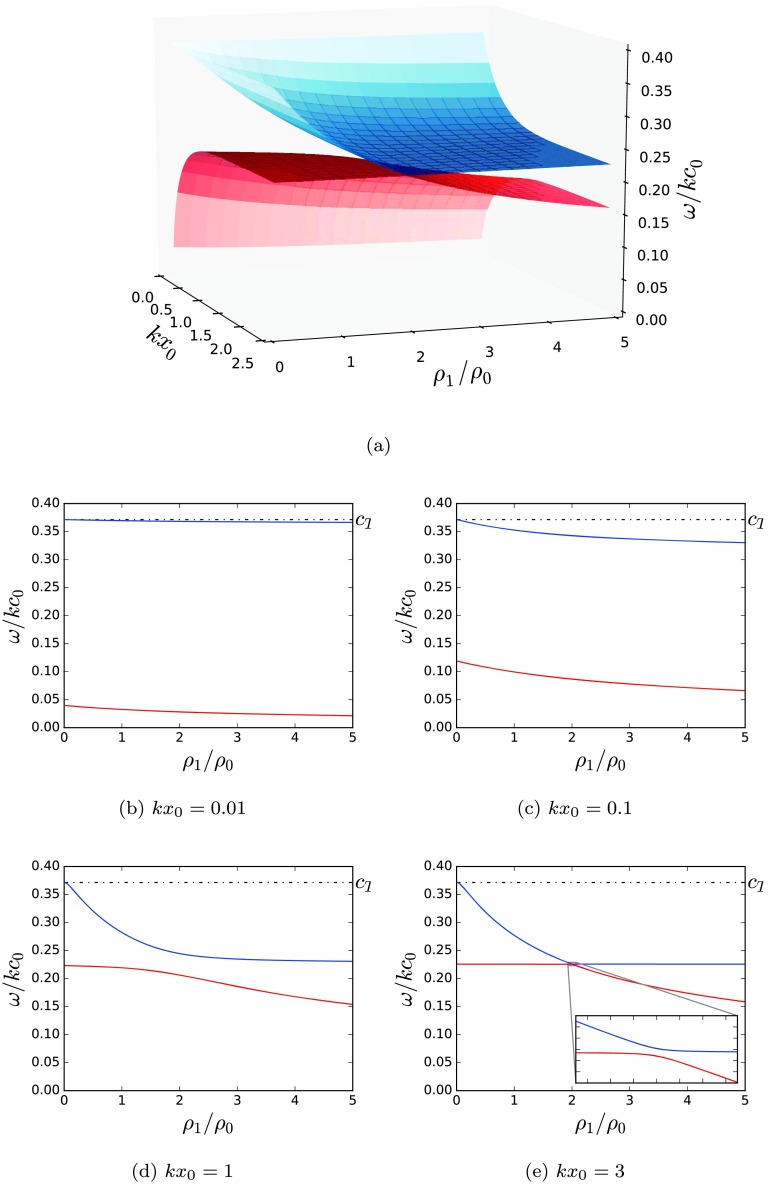
(a) The slow quasi-sausage (blue) and quasi-kink (red) surface mode solutions of the dispersion relation (Equation (20)) are plotted showing the variation of the dispersion as the ratio of one external density to the internal density is changed. The other density ratio is held fixed at $\rho_{2}/\rho_{0}=2$. The characteristic speed orderings are $c_{2}=0.7c_{0}$, $v_{\mathrm{A}}=0.4c_{0}$, and $c_{1}$ varies to satisfy equilibrium pressure balance, given by Equation (3). Figures (b) – (e) are slices of panel (a) for specific values of the non-dimensional slab width, $kx_{0}$.

Figures 7a – 7e illustrate the behaviour of the slow surface modes as the external density on one side of the slab is varied while holding fixed the other external density. The slice where $\rho_{1}/\rho_{0}=2$ corresponds to a symmetric slab, where the usual behaviour is observed: the phase speed of the two slow surface modes converges to a speed that is slower than the tube speed, $c_{\mathrm{T}}$, as the slab width increases. However, as the external densities become distinct, the phase speeds of these modes become distinct for a wide slab. This can also be seen in Figures 5a and 5b.

For a wide slab width, $kx_{0}\gg1$, Figure 7e illustrates that the eigencurves of the slow surface modes demonstrate a wave phenomenon known as “avoided crossing”. Avoided crossings occur when the phase speeds of two wave modes avoid crossing when a parameter of the system is varied due to constraints preventing them from being equal; it demonstrates a transferral of properties between the two modes and can be used to give insight into the modal structure. There is rich literature regarding avoided crossings for the eigensolutions of a wide range of physical processes including energy level repulsion in quantum physics (Naqvi and Brown, 1972) and coupled spring oscillations in classical mechanics (Novotny, 2010); in MHD the subject has been covered only briefly, for example between fast and slow magneto-acoustic gravity waves in a magnetically stratified plasma by Abdelatif (1990) and Mather and Erdélyi (2016).

**Figure 8:**
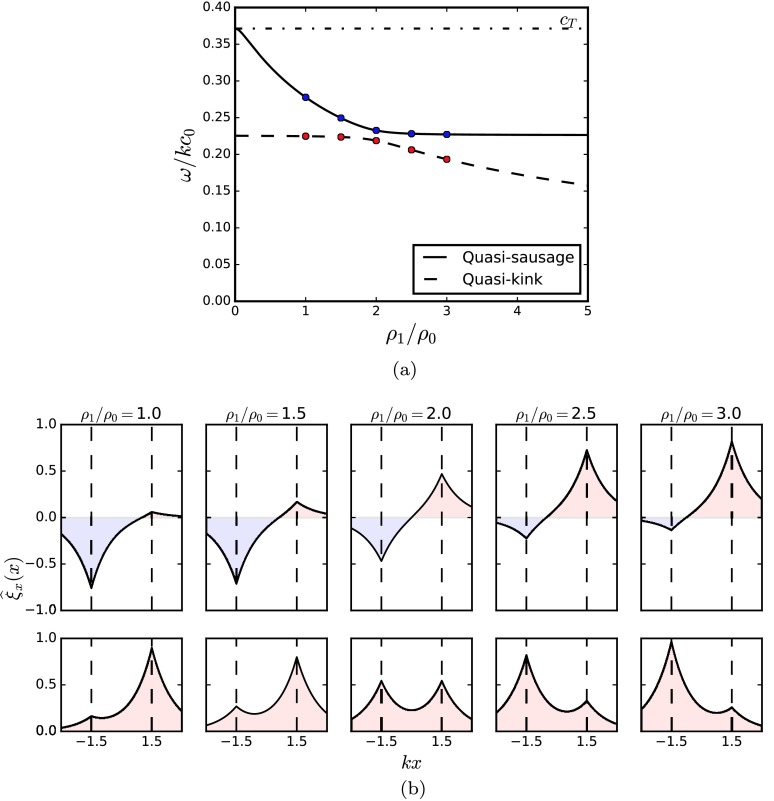
(a) The slow surface mode solutions of the dispersion relation, Equation (20), are plotted showing the variation of the dispersion as the ratio of one external density to the internal density is changed. The other density ratio is held fixed at $\rho_{2}/\rho _{0}=2$ and the non-dimensionalised slab width $kx_{0} = 1.5$. The characteristic speed orderings are $c_{2}=0.7c_{0}$, $v_{\textrm {A}}=0.4c_{0}$, and $c_{1}$ varies to satisfy equilibrium pressure balance, given by Equation (3). The parameters at each blue and red dot in panel (a) are used to plot the spatial variation of the transverse displacement perturbation, $\widehat{\xi }_{x}$, given by panel (b). The upper (lower) plots in panel (b) correspond to the quasi-sausage (quasi-kink) mode solutions.

In the present study, the avoided crossing occurs between quasi-kink and quasi-sausage surface solutions to the asymmetric slab. This explains why the dispersion relation does not decouple into a sausage and a kink equation (Section 2.1). Figures 8a and 8b demonstrate that during the transition across the avoided crossing, the quasi-sausage and quasi-kink modes exchange the slab boundary upon which the largest perturbation occurs. For example, the left plots of Figure 8b show that the quasi-sausage mode has its highest amplitude on the interface of highest local phase-speed (equivalently, lowest external density). The quasi-kink mode demonstrates the opposite behaviour. The central plots show the special case of a symmetric slab, where $\rho_{1} = \rho _{2}$, demonstrating the spatial antisymmetry and symmetry in the symmetric sausage and kink mode, respectively. As the left external density, $\rho_{1}$, dominates the right external density, $\rho _{2}$, the right plots of Figure 8b show that again the quasi-sausage mode has its higher amplitude on the interface of higher local phase-speed, but this is now on the other interface. By the term local phase speed we are referring to the phase-speed of a slow surface mode travelling parallel to the magnetic field at an isolated density and magnetic interface between a magnetised and non-magnetised plasma, *i.e.* half of the magnetic slab model. Each interface of an asymmetric magnetic slab has a distinct “local” phase-speed, and according to Roberts (1981a), these phase speeds are inversely proportional to the density in the non-magnetic region.

When they exist, the fast quasi-sausage and quasi-kink surface modes demonstrate an identical behaviour (not shown). As demonstrated analytically in Equations (34) – (35) and (37) – (40), the body modes are not dependent on internal or external densities to leading order in $kx_{0}$. This means that body modes demonstrate only a weak dependence on the external densities, and an avoided crossing does not occur between these modes as the external densities are varied.

## Discussion

For the first time, a mathematical model of an isolated magnetic slab in an asymmetric environment has been presented, generalising the classic symmetric magnetic slab model (Roberts, 1981b). Key analytical results demonstrate the fundamental differences between the behaviour of linear waves propagating along asymmetric and symmetric slabs.

Sausage and kink modes are traditionally thought of as the fundamental modes of symmetric magnetic slab and tube geometries. Unlike that of a symmetric slab, the dispersion relation governing linear waves along a magnetic slab in a non-magnetic asymmetric external environment does not decouple into two equations, which signifies that the eigenmodes of an asymmetric slab are not the pure sausage and kink modes that we are familiar with; instead, they are adjusted by the asymmetry in the external region and demonstrate mixed properties. For example, the quasi-kink mode does not have a spatially constant perturbed slab width like the symmetric kink mode.

**Figure 9:**
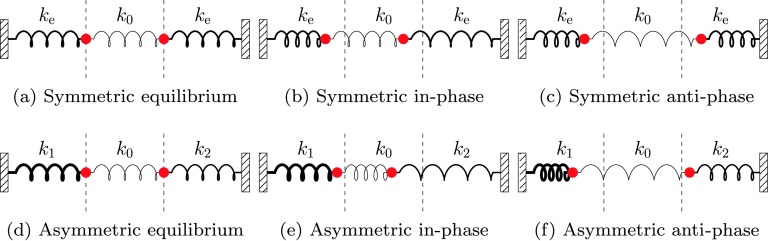
A coupled mechanical oscillator gives an analogy to the eigenmodes of symmetric and asymmetric magnetic slabs. Spring constants are denoted $k$, with a thicker spring corresponding to a higher spring constant. Figures (a) and (d) show the symmetric and asymmetric spring systems in equilibrium. Figures (b) and (c) show the normal modes of a symmetric system. Figures (e) and (f) show the normal modes of an asymmetric system with spring constants $k_{1}>k_{2}$. In each panel, the vertical dashed lines give the positions of the red masses at equilibrium.

To understand this further, we propose an analogy with a coupled mechanical simple harmonic oscillation system (more information can be found in Novotny, 2010). Consider a system of two identical masses, between two fixed walls, with springs connecting the left wall to the left mass, the masses together, and the right mass to the right wall (Figure 9a). The three springs correspond to the three regions of plasma, and the masses to the interfaces. If the left and right springs have the same spring constant, then the two normal modes of oscillation are the oscillations where the masses are in phase (Figure 9b) or in anti-phase (Figure 9c). These are analogous to the kink and sausage surface modes, respectively, in a symmetric magnetic slab. Consider now the case when the spring constants in the left and right springs are distinct (Figure 9d). In this case, the normal modes are asymmetric in-phase (Figure 9e) and in-anti-phase oscillations (Figure 9f), analogous to the asymmetric quasi-kink and quasi-sausage magnetic slab modes, respectively. In this mechanical analogy, it is clear that for the in-phase mode, the higher amplitude will be on the side with smaller spring constant, and *vice versa* for the in-anti-phase mode. A higher spring constant in the left or right springs in this analogy corresponds to a lower density outside the magnetic slab because a higher spring constant in an uncoupled spring gives a higher characteristic frequency. This gives us some motivation as to why the surface modes of the asymmetric magnetic slab have higher amplitudes on different sides for quasi-sausage and quasi-kink modes.

Mixed properties and mode coupling of asymmetric MHD waves are very interesting for solar physics because they signify interaction between different modes, and interaction can mean energy transfer. This indicates a potential plasma heating mechanism: waves with negligible dissipation are excited in the solar interior and propagate through the photosphere and chromosphere until they interact and transfer energy to modes with rapid dissipation in the upper solar atmosphere, *i.e.* corona, where the kinetic and magnetic energy of the wave is converted into thermal energy (Priest, 2014).

There is potential use of the ideas presented here as a diagnostic tool in the emerging field of solar magneto-seismology. Without loss of generality, consider a magnetic slab of plasma in an asymmetric environment with a higher density in the left external plasma than on the right (*e.g.* Figures 2a and 2b). The ratio between the amplitudes of oscillation on the left and right boundaries will be a function of the equilibrium and wave parameters of the slab. This ratio and several other parameters such as the slab width, the wave frequency, and the wavelength are directly observable, and parameters such as the density and temperature in each region can be determined through emission spectra, leaving only the magnetic field parameters unknown. Thus, in the context of solar observations, the cross-slab amplitude ratio could be used to calculate difficult-to-measure solar parameters such as the Alfvén speed and magnetic field strength when observing MHD waves in slab-like structures.

A similar solar magneto-seismological tool that shows its potential is the novel anti-node shift method, which uses the shift due to density structuring of the anti-nodes of standing modes to deduce the parameters of solar environments (Erdélyi and Verth, 2007; Verth *et al.*, 2007; Erdélyi, Hague, and Nelson, 2014). The success of this method indicates the need in the solar physics community for novel solar magneto-seismology methods.

There are clear future generalisations to the asymmetric slab model presented here. The consideration of a constant, uniform shear flow in one or both external environments would generalise the symmetric slab with shear flow studied by Li, Habbal, and Chen (2013), and introduce the additional physics of the Kelvin Helmholtz instability and negative energy waves. This would allow for better application of the asymmetric slab model to dynamic solar environments such as the solar wind, where magnetic structures with flows have been modelled using tube and symmetric slab geometries (Parker, 1965; Nakariakov, Roberts, and Mann, 1996; Taroyan and Erdélyi, 2002; Ruderman, 2010; Terradas *et al.*, 2011; Yu *et al.*, 2016). The generalisation of including a uniform external magnetic field and gravity, either in the direction parallel or perpendicular to the slab, would improve the applicability of the model, at the cost of analytic difficulty. With the completion of the next generation of solar telescopes, including the eagerly awaited Daniel K. Inouye Solar Telescope (DKIST), observations of fine-scale solar magnetic structures look forward to significant improvements in both spatial and temporal resolution; the future looks bright for solar magneto-seismology.
